# Rapid Growth of Lung Nodules due to Combined Pulmonary Vasculitis, Silicoanthracosis, and Chondrocalcinosis

**DOI:** 10.1155/2016/9254374

**Published:** 2016-07-10

**Authors:** Wolfgang Jungraithmayr, Stefanos Tzafos, Oliver Distler, Antonios G. A. Kolios, Walter Weder, Daniel Franzen

**Affiliations:** ^1^Division of Thoracic Surgery, University Hospital Zurich, Raemistr. 100, 8091 Zurich, Switzerland; ^2^Department of Rheumatology, University Hospital Zurich, Raemistr. 100, 8091 Zurich, Switzerland; ^3^Department of Immunology, University Hospital Zurich, Raemistr. 100, 8091 Zurich, Switzerland; ^4^Division of Pulmonology, University Hospital Zurich, Raemistr. 100, 8091 Zurich, Switzerland

## Abstract

*Background.* Silicoanthracosis is a pneumoconiosis due to occupational inhalation of silica and carbon dusts. Clinically, it can be associated with vasculitis or rheumatoid arthritis. In association with these diseases, silicoanthracosis can present within the lung with multiple pulmonary nodules which, as a differential diagnosis, can mimic metastatic disease or multiple abscesses.* Case Presentation. *We present the case of a 62-year old former pit worker with pulmonary nodules, chondrocalcinosis due to calcium pyrophosphate deposition (CPPD), and a history of renal cancer. Within a short period of time, pulmonary nodules grew rapidly. Thoracoscopically, the resected lung specimen revealed silicoanthracosis associated with small-to-medium-size vasculitis in the presence of antineutrophil cytoplasmatic autoantibodies (c-ANCA).* Conclusion. *Pulmonary silicoanthracotic lesions on the base of ANCA-associated vasculitis and CPPD arthritis can rapidly grow. A mutual correlation between silicoanthracosis, ANCA-associated vasculitis, and CPPD seems possible. Apart from this, consideration of metastatic disease should be obligatory in patients with a history of cancer at the same time being immunosuppressed.

## 1. Background

Silicoanthracosis (SA) is a pneumoconiosis derived from accumulation of carbon and silica in the lungs from inhaled coal dust; the silica content can produce fibrous nodules throughout the body [1]. Histology shows central necrosis with giant cell infiltration or classical silicotic nodules. Patients occasionally suffer from dry cough and progressive lung function decline. However, the course of the disease can vary in the context of other diseases. There is evidence that silicosis can induce various forms of autoimmune diseases such as systemic sclerosis and rheumatoid arthritis (RA) [2]. Also, recent data suggest that silicosis increases the risk of development of anticytoplasmatic neutrophilic antibody- (ANCA-) associated vasculitis [3].

This case describes a patient with the unusual course of ANCA-positive vasculitis associated with silicosis and preexisting calcium pyrophosphate deposition disease (CPPD). Particularly the sudden outbreak of clinical symptoms with the development of multiple new lung nodules additionally underlines the significance of important differential diagnoses of multiple pulmonary nodules in the context of arthritis and with a history of cancer.

## 2. Case Presentation

**Figure 1:**
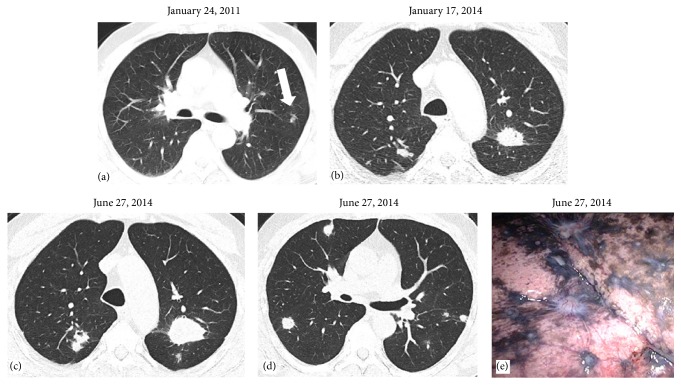
Representative computed tomography (CT) sections of the chest. The CT scan from January 2011 reveals few and small nodules, of which one is wedge-resected from the posterior upper lobe (a, arrow). Three years later, nodules became larger and spread to both sides of the lung (b). CT scans half a year later show a massive increase in size and number but also change of morphology in bilateral lung lesions (c, d). Representative image of the surface from the lower lobe of the right lung, taken during thoracoscopy: white, star-shaped lesions appear retracted into the lung tissue, reminiscent of lung metastases (e).

A 62-year-old male patient presented in January 2011 with cough and hemoptysis, microhematuria, polyarthritis of both ankles, knees, and left wrist, bilateral lymphadenopathy, and pulmonary nodules revealed by a CT-scan (Figure 1(a)). This nonsmoker had worked from 1974 to 1986 in a uranium mining being exposed to silica dusts. His ANCA titer was 1 : 640 with a specificity of PR3-ANCA at 193 U/mL. Neither rheumatoid factors nor anticyclic citrullinated peptide (CCP) was detectable. Although a kidney biopsy was inconclusive, the diagnosis of granulomatosis with polyangiitis was suspected, and cortisone treatment resulted in an amelioration of symptoms. A thoracoscopically resected pulmonary lesion from the left upper lobe (Figure 1(a), arrow) revealed necrotic granuloma with centrally located silicoanthracosis consistent with silicosis. No vasculitis was found in lung or kidney at that time. In February 2011, the patient had a resection of a clear cell carcinoma in the upper pole of the right kidney (pT1 cN0 cM0 G2). Meanwhile, methotrexate and nonsteroidal antirheumatic drugs (NSAR) were added due to ongoing and immobilizing polyarthritis, leading to a rapid and significant improvement of inflammation and pain. While ANCA titers gradually decreased over time to 1 : 40, and PR3-ANCA titers almost normalized, antinuclear antibodies showed a constant level of 1 : 80. However, complement factors and also IgG levels showed normal values throughout the entire clinical course.

**Figure 2:**
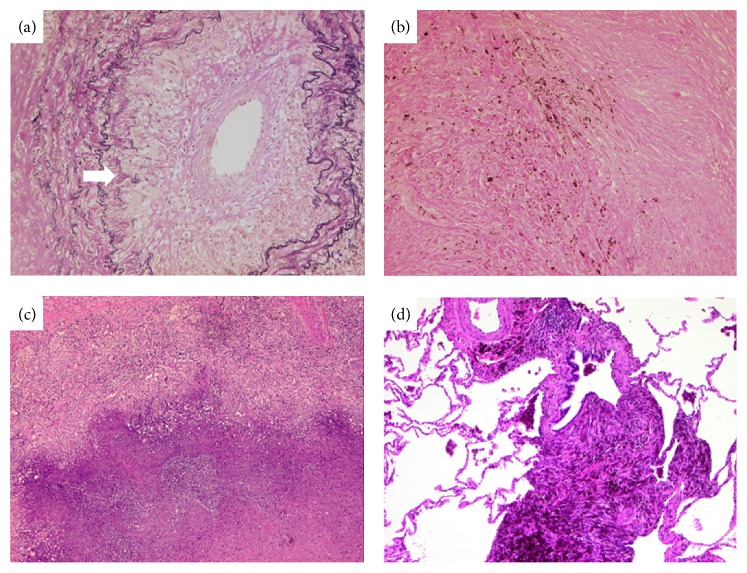
Representative images of histology shows anthracosilicotic dust bands without necrobiotic granulomas and surrounding perivascular fibrotic dust deposition ((b)–(d), magnification 100x). In addition, giant cell vasculitis with fragmentation of the internal lamina elastic wall is seen ((a), arrow) (magnification 200x).

A follow-up thoracic CT scan (January 2014) revealed new bilateral multiple round lesions (Figure 1(b)). Histological specimens provided by transbronchial forceps biopsy could only reveal fibrotic areas with anthracosis and birefringent crystals by compensated polarized light microscopy, corroborated by the aforementioned pathological diagnosis of anthracosilicosis. During the following short time period of 6 months, the patient developed an increasingly nagging cough with repeated expectorations of putrid and musty material several times a day. A CT scan (June 2014) showed a massive bilateral progression of nodules in size and number (Figures 1(c) and 1(d)), further aggravating the patient's symptoms. A bronchoscopic forceps biopsy with radial endobronchial ultrasound (R-EBUS) under fluoroscopic guidance from the largest lesion (Figure 1(c)), but also from other lesions, was performed; however, these biopsies were not diagnostic. Therefore, three representative nodules of the right lung were thoracoscopically resected and appeared as whitish, star-shaped lesions on the pleural surface, highly suggestive of metastases (Figure 1(e)). Inside, they showed a white-greyish surface with dark spots and of crumbly consistency. Histology confirmed anthracosilicotic dust bands, regional necroses, and vasculitis. However, there were no necrobiotic granulomas (Figures 2(a)–2(d)). As a new finding, vasculitis with fragmentation of the internal lamina elastic wall was found (Figure 2(a), arrow), and therapy with rituximab was started. In parallel, therapy with NSAR and methotrexate was stopped, leading to reoccurrence of intolerable joint pain and polyarthritis within several weeks. X-rays of the hands showed hook-like osteophytes in the metacarpophalangeal (MCP) joints, cartilage calcification, and joint space narrowing and subluxation of MCP joints but no RA typical features such as joint erosions. These findings were highly suggestive of calcium pyrophosphate crystal deposition (CPPD) disease as a cause of the patient's polyarthritis. The patient was restarted on NSAR and methotrexate and improved rapidly. At a follow-up visit in October 2015, the patient reported a clear improvement of the polyarthritis and respiratory symptoms.

## 3. Discussion

Growing evidence suggests that silica exposure may induce autoimmune diseases by T-cell activation such as RA, Sjögren's syndrome, sarcoidosis, systemic sclerosis, or ANCA-associated vasculitis [4]. The herein presented patient suffered from ANCA-positive vasculitis and chondrocalcinosis possibly associated with SA.

Two lessons can be learned from this case. First, a rapid progression of pulmonary nodules can be due to the ANCA-positive vasculitis itself but might be caused by other diseases. Since the patient's presentation was unusual due to the fast appearance of symptoms and rapid growth of pulmonary nodules within a few months compared to the slow and mild course before, a malignant cause such as metastases was considered in first instance, particularly considering the positive history of cancer and being under immunosuppressive therapy. Another important aspect why pulmonary nodules rapidly increased in size and morphology is the intake of methotrexate, which is thought to exacerbate rheumatoid nodule formation in selected patients with RA despite the suppression of synovial inflammation [5]. This activity may be mediated via the agonist stimulation of adenosine A1-receptors by methotrexate thereby leading to enhanced giant cell formation [5]. Notably, the increasingly musty expectoration and sputum by the patient could have been also highly suggestive of tuberculosis or intrapulmonary abscesses, in particular in the presence of immunosuppression.

The second lesson from this case is the complex differential diagnosis of patients with SA and the occurrence of polyarthritis. Therefore, Caplan syndrome (CS) was initially suspected. The possible diagnosis could have matched the symptoms and radiological presentation [6]. However, ANCA-associated vasculitis within newly developed lung nodules was found, a feature that is not reported in the context of CS. By contrast, there are several reports on a probable causative relationship between SA and ANCA-associated vasculitis [7, 8]. In addition, the combination of the characteristic X ray changes (cartilage calcification, hook-like osteophytes), the response to NSAR, and the rapid worsening of symptoms after stopping NSAR strongly suggested the additional diagnosis of CPPD disease.

This case presentation reflects how heterogeneous the cause of rapidly growing, symptomatic multiple lung nodules in a complex clinical scenario can be. On the one hand, there seems to be a causative relationship if not a mutual correlation between silicosis and ANCA-associated vasculitis and possibly CPPD disease. On the other hand, given the immunosuppressive state of this patient and a positive history of cancer, lung metastases strongly need to be taken into consideration as an important differential diagnosis.

## Abbreviations

SA: Silicoanthracosis
CPPD: Calcium pyrophosphate deposition
CS: Caplan syndrome
RA: Rheumatoid arthritis
RF: Rheumatoid factor
ANCA: Anticytoplasmatic neutrophilic antibody.

## Additional Points

*Learning Objectives.* Learn that pulmonary nodules secondary to the patients ANCA-positive vasculitis can grow rapidly and can be heavily symptomatic. At the same time, it can mimic metastatic disease, particularly in the presence of immunosuppression and a history of cancer.

*Pretest*. (i) Why do pulmonary nodules on the base of ANCA-positive vasculitis burst so rapidly? (ii) How can pulmonary lesions be distinguished radiologically from metastatic disease?

*Posttest*. (i) Why do pulmonary nodules burst so rapidly? The rapid progression of pulmonary nodules described can be due to the ANCA-positive vasculitis itself. On the other hand, the intake of methotrexate, which is thought to exacerbate rheumatoid nodule formation in selected patients with RA, can exacerbate these pulmonary lesions. A mutual correlation between silicoanthracosis, ANCA-associated vasculitis, and possibly CPPD is most likely causative. (ii) How can pulmonary lesions be distinguished radiologically from metastatic disease? In general, only histologic biopsy, ideally obtained by thoracoscopic wedge resection, can differentiate metastatic disease from other diseases. Neither typical dynamic changes nor the form of those pulmonary lesions allows for a reliable distinction.
